# A model-based clustering method to detect infectious disease transmission outbreaks from sequence variation

**DOI:** 10.1371/journal.pcbi.1005868

**Published:** 2017-11-13

**Authors:** Rosemary M. McCloskey, Art F. Y. Poon

**Affiliations:** 1 BC Centre for Excellence in HIV/AIDS, Vancouver, British Columbia, Canada; 2 Department of Pathology and Laboratory Medicine, Western University, London, Ontario, Canada; 3 Department of Microbiology and Immunology, Western University, London, Ontario, Canada; 4 Department of Applied Mathematics, Western University, London, Ontario, Canada; Temple University, UNITED STATES

## Abstract

Clustering infections by genetic similarity is a popular technique for identifying potential outbreaks of infectious disease, in part because sequences are now routinely collected for clinical management of many infections. A diverse number of nonparametric clustering methods have been developed for this purpose. These methods are generally intuitive, rapid to compute, and readily scale with large data sets. However, we have found that nonparametric clustering methods can be biased towards identifying clusters of diagnosis—where individuals are sampled sooner post-infection—rather than the clusters of rapid transmission that are meant to be potential foci for public health efforts. We develop a fundamentally new approach to genetic clustering based on fitting a Markov-modulated Poisson process (MMPP), which represents the evolution of transmission rates along the tree relating different infections. We evaluated this model-based method alongside five nonparametric clustering methods using both simulated and actual HIV sequence data sets. For simulated clusters of rapid transmission, the MMPP clustering method obtained higher mean sensitivity (85%) and specificity (91%) than the nonparametric methods. When we applied these clustering methods to published sequences from a study of HIV-1 genetic clusters in Seattle, USA, we found that the MMPP method categorized about half (46%) as many individuals to clusters compared to the other methods. Furthermore, the mean internal branch lengths that approximate transmission rates were significantly shorter in clusters extracted using MMPP, but not by other methods. We determined that the computing time for the MMPP method scaled linearly with the size of trees, requiring about 30 seconds for a tree of 1,000 tips and about 20 minutes for 50,000 tips on a single computer. This new approach to genetic clustering has significant implications for the application of pathogen sequence analysis to public health, where it is critical to robustly and accurately identify clusters for the most cost-effective deployment of outbreak management and prevention resources.

## Author summary

Many pathogens evolve so rapidly that they accumulate genetic differences within a host before becoming transmitted to the next host. Consequently, clusters of sampled infections with nearly identical genomes may reveal outbreaks of recent or ongoing transmissions. There is rapidly growing interest in using model-free genetic clustering methods to guide public health responses to epidemics in near real-time, including HIV, Ebola virus and tuberculosis. However, we show that current methods are relatively ineffective at detecting transmission outbreaks; instead, they are predominantly influenced by how infections are sampled from the population. We describe a fundamentally new approach to genetic clustering that is based on modelling changes in transmission rates during the spread of the epidemic. We use simulated and real pathogen sequence data sets to demonstrate that this model-based approach is substantially more effective for detecting transmission outbreaks, and remains fast enough for real-time applications to large sequence databases.

## Introduction

Genetic clustering is a class of methods for reducing large sequence data sets down to groups of closely-related sequences. In the context of infectious diseases, clusters may identify infections related by a common source [1]. Additionally, genetic clusters may represent locally elevated rates of transmission, particularly when we expect a measurable number of genetic differences to accumulate within a host between transmission events. Because genetic sequencing is increasingly a fixture of the clinical management of infections, there is growing interest in making secondary use of the resulting databases as a cost-effective resource for guiding public health responses in near real time [2–4]. The general motivation is that if genetic clusters define groups with higher rates of transmission, then they may facilitate a more cost-effective deployment of prevention services.

A diverse number of genetic clustering methods have been developed and applied for a broad range of bacteria and viruses including *Staphylococcus aureus* [5], *Mycobacterium tuberculosis* [6], HIV [7, 8], hepatitis C virus [9, 10] and Ebola virus [11, 12]. These clustering methods are nonparametric because the clustering criteria are based on empirical distributions, making no specific assumptions about the underlying biological processes. For instance, pairwise methods build up clusters from pairs of sequences with a genetic distance below a predefined threshold [5, 13]. Subtree methods define clusters relative to the common ancestors of sequences in the phylogenetic tree, based on quantities such as the mean branch length among the descendants of the ancestral node [14, 15]. Nonparametric methods tend to be intuitive and relatively easy to compute. We have previously observed, however, that current nonparametric clustering methods seem relatively insensitive to variation in rates of transmission [16]. Instead, these methods tend to detect variation in rates of sampling, *i.e*., the delay between infection and diagnosis.

Here we introduce a fundamentally new approach to genetic clustering for infectious diseases that is based on modelling the evolution of transmission rates along the tree. We demonstrate that our model-based (parametric) clustering method substantially outperforms a variety of nonparametric methods in recovering clusters of rapid transmission in simulated data, and we show that these differences are recapitulated in an analysis of real HIV-1 data.

## Methods

As in previous work [17, 18], we assume that the phylogenetic tree reconstructed from the genetic variation among sampled infections is similar in shape to the underlying transmission tree [19]. Hence, we assume that a branching point in the tree roughly approximates a transmission event, which is also an implicit assumption of nonparametric clustering methods using trees, where subtree clusters are interpreted as ‘hotspots’ of rapid transmission. We model branching rates as a discrete character state that evolves along a phylogeny according to a continuous-time Markov chain [20]. Further, we assume the branching events in the phylogeny are generated according to a Poisson process whose rate is controlled by the evolving character. Under this model, the branch lengths of the phylogeny are not independent of the character state, as typically assumed when modeling the evolution of nucleotides or amino acids.

A Cox process, or doubly-stochastic Poisson process, is an inhomogeneous Poisson process whose arrival rate λ(*t*) is itself a stochastic process. The Markov-modulated Poisson process (MMPP) is a special case of the Cox process where λ(*t*) varies according to a continuous-time Markov chain with a finite number of states. When the Markov chain is in state 1, the Poisson process has rate λ_1_, and so on. Following [21], we will let the Markov chain have *m* states, denote its infinitesimal generator matrix by:

$$\begin{array}{cc} Q & =[\begin{array}{cccc} -\sigma_{1} & \sigma_{12} & \cdots & \sigma_{1m}\\ \sigma_{21} & -\sigma_{2} & \cdots & \sigma_{2m}\\ \vdots & \vdots & \ddots & \vdots \\ \sigma_{m1} & \sigma_{m2} & \cdots & -\sigma_{m} \end{array}] \end{array}$$

where *σ*_*i*_ = ∑_*j*≠*i*_ *σ*_*ij*_, and denote the vector of rates of the Poisson process by λ = [λ_1_, …, λ_*m*_]^*T*^.

The probability density of the process producing its first arrival at time *y* in state *j*, given that it started in state *i* at time 0, is the *ij*th element of the matrix *f*(*y*) = exp((*Q* − Λ)*y*)Λ where Λ = diag(λ) [22]. Following [23] and [24], it is straightforward to calculate the likelihood of an observed tree under this process. Let *v* be an internal node of the *τ* other than the root, *u* be its parent, *w* and *z* be its children, and *t*_*v*_ be the length of the branch joining *v* to its parent *u*. As in [24], define *L*_*i*_(*v*) to be the likelihood of the subtree rooted at *v* conditioned on the parent *u* being in state *i*. *L*_*i*_(*v*) is recursively defined by

$$L_{i}\left(v\right)=\sum_{j}\;f_{ij}\left(t_{v}\right)L_{j}\left(w\right)L_{j}\left(z\right).$$

At the root of the tree, with children *w* and *z*,

$$\begin{array}{ccc} L_{i}\left(\tau \right) & = & \pi_{i}L_{i}\left(w\right)L_{i}\left(z\right)\\ L(\tau ) & = & \sum_{i}\;L_{i}\left(\tau \right). \end{array}$$

At a tip node *v* with branch length *t*_*v*_, it is intuitive to define the likelihood using the matrix exp((*Q* − Λ)*t*_*v*_), which gives the probability density of each state transition and no events occurring up to time *t*_*v*_. However, we found that the parameters which optimized the likelihood with this definition nearly always included one arbitrarily small rate assigned to all tips. For this reason, we simply assigned *L*_*i*_(*v*) = 1 for all tips *v*. This approach is likely to overestimate cluster sizes due to inclusion of non-cluster individuals sampled following transmission from a cluster member, as well as individuals who are not currently part of a cluster but were in the past. To optimize the likelihood of this model, we used the covariance matrix adaptation evolution strategy [25], a black-box, derivative-free optimization algorithm.

To generate clusters from the fitted MMPP model, we reconstructed the maximum likelihood character states ($\hat{\lambda }$) at the ancestral nodes of the tree using the joint maximum likelihood algorithm formulated by [24]. Taking the lowest rate class as the background state λ_0_, we extracted all contiguous subset trees comprising ancestral nodes with $\hat{\lambda }>\lambda_{0}$ using a pre-order traversal algorithm. Source code implementing the MMPP clustering method is available under the GNU General Public License (version 3.0) at https://github.com/rmcclosk/netabc (executable *pcbr*).

### Data simulation

Trees were simulated using MASTER (version 5.0.2 [26]) under a susceptible-infected-removed (SIR) model of an epidemic, as described in previous work [16]. In brief, transmissions occur between infected (*I*) and susceptible individuals (*S*) at a rate *βSI*. Individuals are removed from *I* due to mortality at a rate *μ* or by becoming sampled at a rate *ψ*. The population is structured into two subpopulations with constant migration between like compartments (*S*_0_ ↔ *S*_1_, *I*_0_ ↔ *I*_1_) at a rate *m*. The two subpopulations comprised *S*_0_ + *I*_0_ = 9000 and *S*_1_ + *I*_1_ = 1000 individuals, respectively. Each epidemic was seeded by a single infected individual in the majority subpopulation (*I*_0_ = 1 at time 0). We simulated 100 replicate trees under three different scenarios where rates of transmission (*β*_1_) and sampling (*ψ*_1_) were varied in the minority subpopulation as follows: (1) a faster sampling rate (*ψ*_1_ > *ψ*_0_); (2) a faster transmission rate (*β*_1_ > *β*_0_); or (3) both faster sampling and transmission rates relative to the majority subpopulation. Hence, our model contained two sets of rate classes for each subpopulation. The underlying assumption is that samples from the minority subpopulation should be assigned to clusters. Infected individuals were removed due to mortality at a constant rate *μ*. The end condition for each simulation was for the tree to reach 1000 terminal branches (tips), which were subsequently filtered for tips corresponding to sampled individuals. As a result, the final number of tips was stochastic and slightly less than 1000. The simulation outputs were serialized to files in the Newick tree specification format and parsed using regular expressions in a Python script to transfer node attributes from comment strings to node labels.

In our preliminary study [16], the parameters of the model were manually adjusted until it yielded tree simulations that resembled the typical ‘star-like’ shape of HIV-1 molecular phylogenies (long terminal branches). For instance, S1 Fig compares the distributions of internal and terminal branch lengths, normalized by the mean branch length, for an HIV-1 phylogeny reconstructed from actual data (see next section) and a tree simulated under this set of model parameters. We also ran a second set of simulations under different parameters to evaluate the sensitivity of our results to these settings. Specifically, we reduced the baseline (majority) transmission rate from *β*_0_ = 5 × 10^−3^ to 7.5 × 10^−4^ and reduced the sampling fraction *ψ*/(*ψ* + *μ*)—the probability that an infected individual was sampled before death—from 0.98 to 0.5 to reflect the parameter settings used in [27]. We note that this sampling fraction is very different from the sampled proportion of the infected population, since the former does not include unsampled and surviving infected individuals. Unlike [27], we increased the baseline sampling rate from *ψ*_0_ = 0.15 to 1.0 so that the latter sampled proportion increased from less than 10% to about 40%. When *ψ*_0_ = 0.15, the prevalence tended to exceed 90% at the simulation end-point where the target number of tips (*n* = 2000) was obtained. The parameter settings used in the two sets of simulations are summarized in S1 Table. For each scenario, we discarded a small number of replicates where the epidemic failed to spread and ran additional simulations until 100 replicates were obtained.

Sequence evolution was simulated on each tree using INDELible (version 1.03) [28] with modifications to allow the user to specify the ancestral nucleotide sequence at the root. We initialized our simulations with the HXB2 *pol* reference sequence (Genbank accession K03455.1) at the root of each simulated tree. As previously described [16], the simulation parameters were calibrated to an actual alignment of HIV-1 *pol* sequences. Specifically, we used the M3 codon model with a transition rate bias *κ* = 8.0 and rate variation according to a gamma distribution (shape *α* = 1.5, rate *β* = 3) that we partitioned into 50 rate categories; a Lavalette distribution (LAV) indel model with *a* = 1.5, *M* = 4 and rate 0.001; and a scaling factor of 15.

Phylogenetic trees were reconstructed from the multiple sequence alignments using approximate maximum likelihood (FastTree2, version 2.1.10) [29] or neighbor-joining (RapidNJ, version 2.3.2) [30]. The alignments and reconstructed trees were used as inputs for five published non-parametric clustering methods—HIV-TRACE (TN93) [31], PhyloPart [32], Cluster Picker [8], subtree clustering [15], patristic distance on bootstrapped alignments [33]—and our MMPP method. Because our method requires a rooted bifurcating tree, we randomly resolved polytomies using the *multi2di* function in the R package *ape* [34] and used midpoint rooting with R package *phangorn* [35].

To measure the performance of each method, we calculated the false and true positive rates for a range of threshold parameter settings for each method, where a ‘positive’ prediction is the assignment of a sampled sequence to a cluster. For TN93 [36], we varied the genetic distance cutoff at all observed values. For Cluster Picker [8], we fixed the bootstrap threshold to 0.9 and varied the distance cutoff from 0.005 to 0.1. Similarly, we varied the maximum distance from 0.005 to 0.0125 in PhyloPart [32]. We implemented a subtree clustering method with Biopython [37], where we screened internal nodes for a bootstrap threshold of 0.9 and varied the mean branch length cutoff from 0.002 to 0.2. Lastly for the bootstrapped patristic method [33], we varied the cutoff from 0.004 to 0.05 for a minimum of 80% of bootstrap replicates.

### Empirical data

To compare these clustering methods on an actual data set, we obtained 3102 published partial HIV-1 *pol* sequences that were previously collected in Seattle, USA, and analyzed for clusters of transmission [38]. We reduced the data down to a single sequence per patient (*n* = 1653) by excluding sequence records that were annotated as an additional isolate (suffixed with an underscore and integer). Next, we removed codons associated with drug resistance mutations according to the surveillance list published by Shafer and colleagues [39], using pairwise alignment of each sequence against the HXB2 *pol* reference to locate the respective codons. This step also trimmed sequence intervals that did not align to the reference. Aligned sequences that were shorter than 100 nucleotides (*n* = 22) were filtered out at this stage. We used SCUEAL [40] to predict HIV subtypes from the sequence data (maximum three recombination breakpoints) and screened for sequences categorized as subtype B excluding intra- and inter-subtype recombinants (*n* = 1653). A multiple sequence alignment was generated from these data using MAFFT (version 7.305b) [41]. We reconstructed a neighbor-joining tree from this alignment with RapidNJ [30] and a maximum likelihood tree with FastTree2 [29]. Finally, we applied the different clustering methods using these trees and, if necessary, the sequence alignment as inputs.

## Results

**Fig 1:**
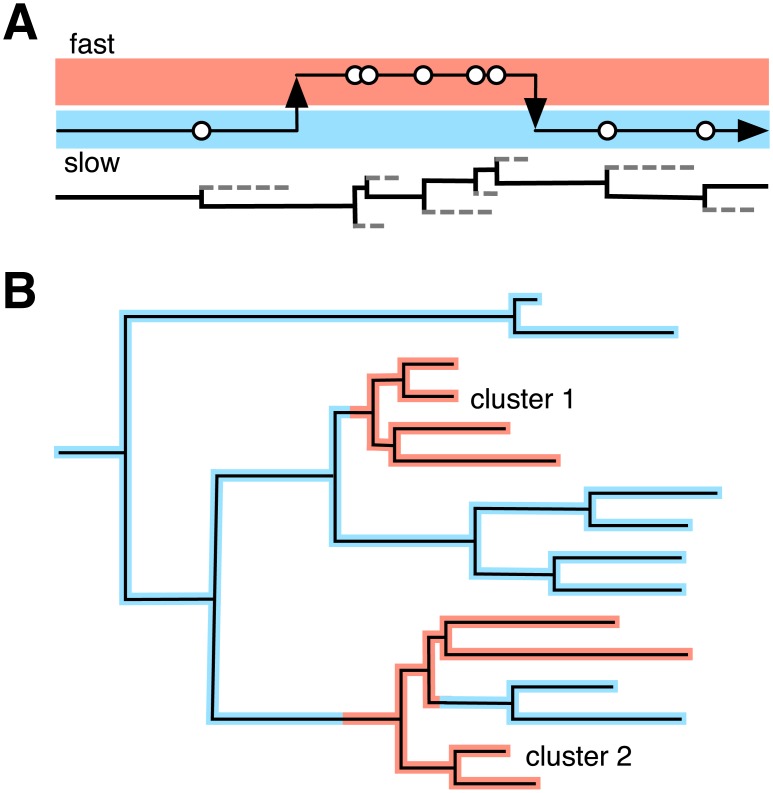
Schematic diagram of a model-based genetic clustering method. The rate of branching events (approximating transmission events) evolves according to a Markov-modulated Poisson process (MMPP). (A) A lineage switches between two states (fast and slow) that control the rates that branching events occur. (B) When rate shifts are mapped to the tree, we can identify clusters of high transmission rates (red).

Epidemic dynamics were simulated from a structured birth-death model using parameter settings from [16]. We assumed that one subpopulation was larger than the other with 9000 and 1000 individuals, respectively, and that the epidemic started with a single infected individual in the larger subpopulation. This model was designed to produce genetic clusters when the epidemic moved from the larger subpopulation into the smaller (minority) subpopulation in which rates of transmission and/or sampling were elevated. With replicate trees generated from the model, we simulated the molecular evolution of viruses along the trees, and then produced sequence alignments and tree reconstructions to use as inputs for five different non-parametric methods. In addition, we analyzed these data using our model-based clustering method that is based on a Markov-modulated Poisson process (MMPP). Put simply, we assume that each branching event in the tree approximates a transmission event. We next assume that these events occur at a constant rate over time, that this rate can switch between a finite number of rate categories, and that these switches in branching rates also occur at a constant rate over time (Fig 1A). The distribution of rate categories throughout the tree can define a partition of branches into clusters (Fig 1B).

**Fig 2:**
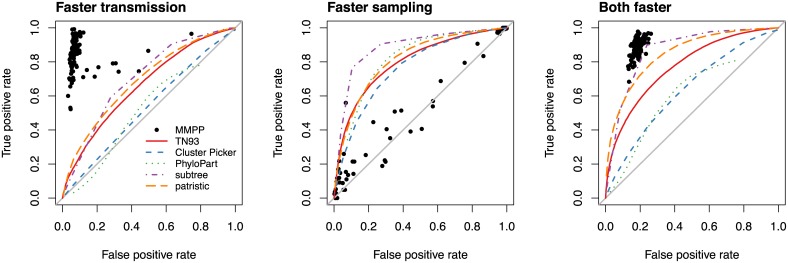
Performance of MMPP and five nonparametric clustering methods on simulated data. Sequence data were simulated under three scenarios where the minority subpopulation had (1) a faster transmission rate (left); (2) a faster sampling rate (centre), or; (3) both faster rates of transmission and sampling (right). The *x*- and *y*-axes correspond to the false and true positive rates of classifying individuals into the minority subpopulation, respectively. Each point represents the outcome when the MMPP model was applied to one of 100 replicate simulations. Each line represents the receiver-operator characteristic curve for one of the five nonparametric clustering methods (see figure legend), where different false and true positive rates were obtained by varying a threshold parameter of the method.

Our main results are summarized in Fig 2 with respect to their true and false positive rates on 100 replicate simulations per scenario. In the first scenario, the minority subpopulation had a 3-fold faster per-lineage transmission rate but the same sampling rate as the majority subpopulation (‘faster transmission’). The MMPP method obtained high true positive rates (TPRs) and low false positive rates (FPRs) with means of 84.6% and 9.2%, respectively. We noticed that a small number (*n* = 7) of replicates resulted in substantially higher FPRs (> 20%) than the others. In these cases, we determined that the MMPP method had incorrectly assigned the ‘faster transmission’ state to the root of the tree. We subsequently determined that increasing the number of rate classes in the model enabled MMPP to correctly identify clusters with the highest rate class. None of the nonparametric clustering methods obtained comparable TPRs or FPRs under this simulation scenario. For instance, the TN93, patristic and subtree clustering methods obtained a mean TPR of about 60% for an FPR of 30%. In contrast, Cluster Picker and PhyloPart performed poorly under this scenario, yielding cluster assignments with TPR and FPR rates that were comparable to random guessing (Fig 2). We obtained similar results when the disparity in transmission rates was increased from 3-fold to 10-fold (S2 Fig).

Furthermore, we verified that the maximum likelihood estimates (MLEs) from the MMPP method were accurately inferring the true transmission rate ratio between the minority and majority subpopulations, and that the accuracy and precision of these estimates increased with sample size (S3 Fig). For trees with 1000 tips, for instance, the interquartile range in ratio MLEs was 2.83 to 3.26, and the mean MLE was 3.10 (the true value was 3.0).

**Fig 3:**
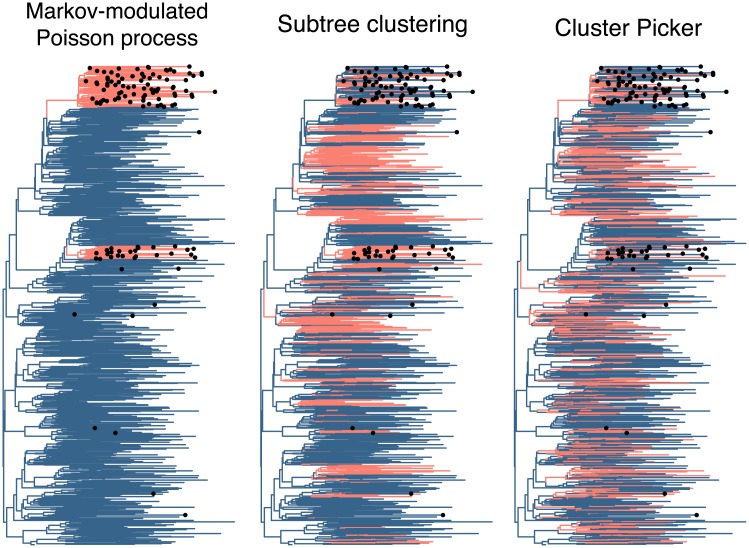
Comparison of predicted clusters and actual clusters of faster transmission. We mapped the clustering predictions from three different methods (Cluster Picker, subtree clustering and MMPP) onto one of the neighbor-joining trees reconstructed from sequence data simulated under the faster-transmission scenario. Branches are coloured light red if the method assigns that branch to a cluster, and dark blue otherwise. The correct assignments are indicated by labeling tips with filled circles if they belong in a cluster. Cluster Picker (version 1.2.4) was run with default initial and main support thresholds (0.9) and a genetic distance threshold of 0.025. Subtree clusters were extracted from the tree with a bootstrap threshold of 90% and mean branch length threshold of 0.0065.

To illustrate the discordant results among these methods, we summarized the cluster assignments for Cluster Picker, subtree clustering and MMPP in Fig 3. In this specific example, sequences sampled from the minority subpopulation are concentrated in two groups. Clusters identified by Cluster Picker were uniformly distributed throughout the tree. In contrast, cluster assignments by the subtree clustering method were distributed less evenly, with one subtree cluster coinciding accurately with one of the actual clusters. However, we found that this method was highly sensitive to the choice of mean branch length cutoff. For instance, increasing this cutoff from 0.0065 to 0.008 caused every terminal branch to be assigned to a cluster.

In the second simulation scenario, the rate of sampling was elevated in the minority subpopulation but the transmission rates were held constant (‘faster sampling’). In other words, members of the minority subpopulation were more likely to be diagnosed sooner after infection. Here, true positives meant correctly selecting tips from the subpopulation with elevated sampling rates. Under this scenario the MMPP predictions were no better than a random guess with a mean TPR and FPR of 55.6% and 51.5%, respectively. This outcome was not surprising given that variation in sampling rates was not a target of the model; *e.g*., terminal branch lengths were excluded from maximum likelihood parameter estimation of the MMPP model. The nonparametric methods were far superior, with the best results obtained by the subtree clustering method. At a bootstrap cutoff of 90% and mean branch length cutoff of 0.006, for example, the average TPR and FPR was 78.8% and 11.9%, respectively.

Finally, the third simulation scenario combined both faster rates of sampling and transmission in the minority subpopulation (‘both faster’). The MMPP predictions attained a mean TPR and FPR of 88.7% and 18.5%, respectively. This level of performance was comparable to the subtree clustering and pairwise distance methods (patristic distance and TN93; Fig 2) at specific cutoff values. On the other hand, the Cluster Picker and PhyloPart methods both suffered worse performance under this scenario.

We obtained qualitatively similar results (S4 Fig) when the trees were simulated under a different parameterization of the birth-death model based on [27]. Relative to the first parameter settings, the transmission rates in both subpopulations were reduced by a factor of 0.15, and the mortality rate of infected individuals was increased to equal the sampling rate in the majority subpopulation. In general, we observed higher FPR associated with the MMPP method for this set of simulations. The mean TPR and FPR of MMPP under the faster transmission scenario were 90.4% and 31.1%, respectively. To our surprise, MMPP was able to correctly identify clusters under the faster sampling scenario (82.3% TPR and 28.8% FPR), unlike the previous set of simulations. This level of performance was comparable to the subtree clustering method. We attribute this difference to the effect of lineage removal due to the elevated mortality of infected individuals [42]. The elevated sampling rate in the minority subpopulation resulted in more complete sampling of the corresponding subtree, leading to shorter lengths of internal branches. In contrast, infected individuals were seldom removed by mortality before sampling under the previous parameterization of the birth-death model.

### Computing time

Based on our simulation analyses, the MMPP method seems to confer greater sensitivity and specificity to detect variation in transmission rates than the five nonparametric methods that we evaluated. These clustering methods are often applied to large sequence databases with thousands or tens of thousands of records [33, 43, 44]. Furthermore, there is a growing demand for genetic clusters to be identified rapidly so that this information can be used to inform public health decisions in near real-time [4, 31]. Hence, we evaluated the average computing time required to extract clusters from simulated data sets containing approximately 1000 sequences each; the actual mean number of tips per tree was 983.3 (range 969–994). Our results, based on the times required to process five replicate data sets, are summarized in Table 1. The most time-consuming method was the patristic distance method because our default approach was to generate distances for 100 nonparametric bootstrap samples of the data [33]. Accordingly, eliminating the bootstrap sampling reduced the computing time by roughly 100-fold at the cost of sensitivity and specificity. The fastest method was TN93, which does not require reconstruction of a phylogenetic tree from sequence variation. Our MMPP method required substantially more time to compute. However, the half-minute used to process a 1000-tip tree is still an acceptable amount for near real-time monitoring.

**Table 1:** Computing time required by six clustering methods to process five different trees each relating approximately 1000 simulated sequences.

Method	Tree required	Multi-core	Time per replicate (seconds)					Average (seconds)
			1	2	3	4	5	
MMPP	yes	no	27.32	31.63	32.54	33.35	27.42	30.45
TN93	no	yes	1.46	1.24	1.21	1.21	1.17	1.26
Cluster Picker	yes	yes	1.45	3.97	2.87	6.20	4.42	3.78
PhyloPart	yes	yes	2.78	3.11	4.64	7.20	6.01	4.75
Subtree clustering	yes	no	2.73	2.69	2.74	2.78	2.83	2.76
Bootstrapped patristic	yes	no	82.46	67.57	95.77	71.71	71.25	77.75

All methods were evaluated on an Intel Xeon E5-1650v4 (six core) processor. If the UNIX time output implied multi-core processing and the use of multiple cores was documented for the program, then this was indicated under the heading ‘Multi-core’. None of the reported times include the time required to reconstruct phylogenetic trees from the simulated sequence data, since the specific reconstruction method used (*e.g*., maximum likelihood, neighbor joining) may vary among users. Because we observed substantial variance among repeated runs of MMPP on the same data, we reported the average of 3 runs. Times reported for TN93 include filtering the genetic distance calculations for the shortest pairwise distance per sequence.

To assess how MMPP computing time scales with the size of the tree, we performed additional experiments on simulated trees with the number of tips varied from 100 to 50,000 tips. These results are summarized in S5 Fig. Our results indicated that the computing time scaled linearly with the size of the tree. For instance, it required about 21.6 minutes on average to process a tree with 50,000 tips. We also observed an apparent ‘phase transition’ in time complexity in the vicinity of about 750 tips (from about 5 to 30 seconds) for the MMPP model with two rate classes. Finally, we repeated these experiments with a 3-rate class MMPP model and obtained similar timings (S5 Fig).

### Application to real data

**Fig 4:**
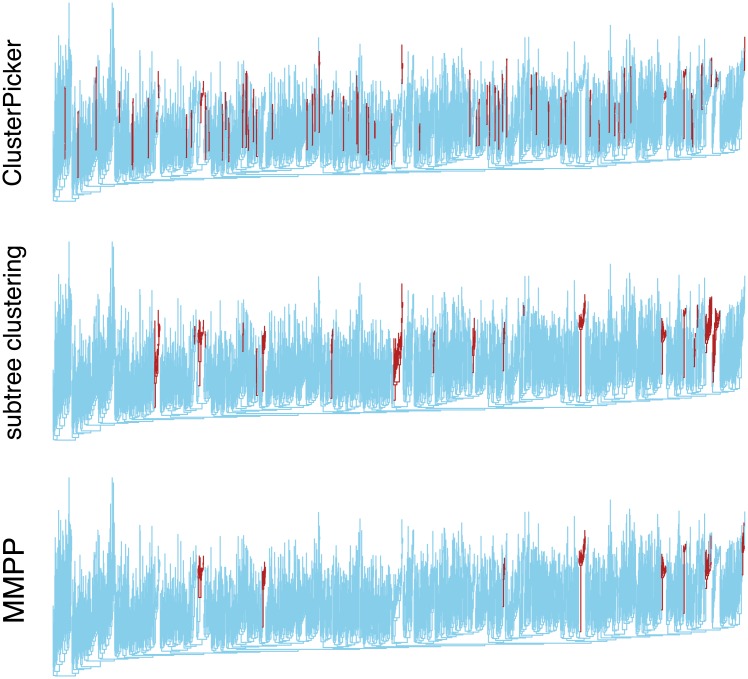
Comparison of three different clustering methods applied to a phylogeny reconstructed from real HIV data. The phylogeny was reconstructed by maximum likelihood from a published data set of HIV-1 subtype B *pol* sequences from a recent study of genetic clusters in Seattle, U.S. [38]. We adjusted the ClusterPicker and subtree clustering method parameters until the number of individuals assigned to clusters was similar to the number reported in the original study. For instance, the subtree method was used with a mean branch length cutoff of 0.65% and a bootstrap cutoff of 90%. Branches assigned to clusters are highlighted in dark red.

We obtained a published data set of *n* = 3102 HIV-1 *pol* sequences that were collected from a study of HIV-1 genetic clusters in Seattle, U.S. [38]. These data were reduced to one sequence per individual and then filtered for non-recombinant subtype B sequences (*n* = 1653, see Methods). We reconstructed a maximum likelihood phylogeny from these sequences as the primary input for the different clustering methods. First, we discovered that the MMPP method tended to assign faster branching rates throughout the base of the tree, including the root node. A lineages-through-time plot of the tree (S6 Fig) was consistent with a period of population-level exponential growth in the first half of the tree, which may confound the MMPP model from detecting other sources of branching rate variation over time. Based on our experience with simulated cases where the faster of two rate classes was sometimes incorrectly assigned to the root of the tree, we increased the number of rate classes to 3 and grouped lineages assigned to the fastest rate class as putative clusters. The resulting clusters are summarized in Fig 4, alongside the clusters predicted by two nonparametric methods (TN93 and subtree clustering). Our MMPP program required 43.6 and 28.7 seconds to analyze the ML tree assuming two and three rate classes, respectively (the slightly faster processing under the 3-rate model was consistent with our timing results for simulations of 1,000-tip trees; S5 Fig). Following the methods described in [38], we initially generated nonparametric clusters using ClusterPicker with a bootstrap cutoff of 95% and a branch length cutoff of 1.5%. However, these settings yielded only 51 clusters of 118 individuals, substantially less than the reported numbers (72 clusters of 168 individuals [38]). This discrepancy was likely caused by minor differences in sequence processing and alignment. We subsequently adjusted the branch length cutoff to 1.8% to obtain 69 clusters of 162 individuals.

The most apparent difference between the nonparametric methods and the MMPP results, which are summarized in Fig 4 and S7 Fig, was the number of individuals assigned to clusters. Our MMPP model assigned only 78 individuals (150 branches in the tree) to 8 clusters, substantially fewer than the number reported in [38]. Based on the assignment of branches to clusters, MMPP was more concordant with the subtree method (Cohen’s *κ* = 0.573) than Cluster Picker (*κ* = 0.101), where *κ* = 1 indicates complete agreement. Within clusters identified by the subtree method but not by MMPP, terminal branch lengths (*n* = 92) were significantly shorter (Wilcoxon test, *P* < 10^−12^) and internal branch lengths (*n* = 64) were no different (*P* = 0.24) from their respective distributions in the entire tree. Similarly, terminal branches in clusters identified by Cluster Picker and not by MMPP were significantly shorter (*n* = 143, *P* < 10^−16^) but not internal branches (*n* = 13, *P* = 0.34); we note the number of branches in the latter comparison was small because the majority of Cluster Picker clusters were dyads. In contrast, internal branches were significantly shorter in MMPP clusters than the rest of the tree (*P* = 1.84 × 10^−7^). As noted in [45], internal branch lengths can provide approximate upper bound estimates of local transmission rates. These results suggest that many of the nonparametric clusters may be caused by variation in sampling rates, which has been anticipated by mathematical modeling [42]. However, we cannot ascertain the extent of this effect without additional information such as estimated dates of infection.

## Discussion

Here we have formulated and tested a model-based (parametric) method for clustering genetic sequences that have been sampled from an infectious disease epidemic. A potential advantage of a model-based approach is that we can focus on the parameter of interest—namely, the variation in transmission rates over time that may be an indicator of outbreaks. Our approach is conceptually similar to the lineages-over-time model proposed by Holmes and colleagues [17]. However, we assume that branching rates evolve along branches of the tree as the epidemic spreads from one risk group to another, such that discordant rates may occur on contemporaneous branches on different parts of the tree. The MMPP model is also closely related to models of biological speciation where rates of speciation are determined by a single evolving character state, *e.g*., [20, 46]. Specifically, our model can be interpreted as a special case of the multitype speciation-extinction model [47] where extinction events are censored from the model. Our simulation results indicate that the interpretation of MMPP clusters with respect to sampling rates is dependent on the probability that a genetic sequence of the pathogen is sampled from an infected individual before their death. If most infected individuals are eventually sampled, then we predict that MMPP clusters will predominantly be determined by variation in transmission rates. However, if a substantial fraction of infected individuals are never sampled before their death and this varies among groups, then the MMPP clusters may be caused by variation in either rates of transmission or sampling. Nevertheless, we submit that this potential confounding is preferable to being unable to detect clusters of transmission at all (S2 Fig).

When interpreting clusters produced under the MMPP model, we assume that the variation in the rates of branching events in the phylogeny is a sufficient approximation of variation in transmission rates over time. This not only assumes that the phylogeny was reconstructed without error, but it also assumes that branching events roughly correspond to transmission events. In general, nonparametric clustering methods that utilize trees require similar assumptions. There are several reasons why transmission events are likely to map to locations of the virus phylogeny other than the branching points, such as incomplete lineage sorting within hosts [48] and incomplete sampling of the infected population [49]. Another issue that is unique to model-based clustering is the problem of model misspecification. The present MMPP model assumes that rates of transition between branching rates are constant over time. For instance, fitting the MMPP model with two rate classes to the actual HIV-1 data set resulted in a majority of branches assigned to the faster rate class, including the root of the tree. This was likely caused by an early period of exponential growth in the epidemic (S6 Fig), which induces lineage-independent variation in branching rates over time. Furthermore, the spread of an epidemic through a socially and spatially-structured host population is problematic for the MMPP model, which assumes that the evolution of branching rates is a memoryless process (S8 Fig). These issues identify directions for further work in this new class of genetic clustering methods for infectious disease outbreaks.

Genetic clustering can be an important resource for retrospective epidemiological investigations [50] and may eventually play a central role in the near-real time monitoring and prediction of infectious disease outbreaks [3, 4]. However, the growing popularity of applying genetic clustering to detect outbreaks of transmission needs to be tempered with greater skepticism about the underlying methods [42, 51]. Our model simulations represent a highly simplified hypothetical scenario where many clustering methods could potentially misdirect public health efforts away from groups suffering from higher rates of transmission, and towards groups where new infections were diagnosed sooner than the population average. Although ‘all models are wrong’, these simulations are instructive because they clarify the respective contributions of variation in sampling and transmission rates to genetic clustering. Furthermore, our results are transferrable to applications of genetic clustering to other infectious diseases where genetic differences can accumulate in the period between infection and sampling. For example, whole-genome clustering analysis is becoming an important resource for tracking outbreaks of *Mycobacterium tuberculosis* [52], which can remain latent for years after infection until reactivation. Hepatitis C virus infections also remain asymptomatic for years after transmission with rapid within-host evolution, and genetic clustering may be confounded with sampling rates [53]. As we have shown with our analysis of the MMPP method, bringing new approaches to these clustering problems may provide a more complete picture, in combination with current methods, about the recent histories of infectious disease epidemics.

## Supporting information

S1 Fig Comparison of internal and terminal branch lengths in simulated and real HIV-1 phylogenies. Histograms summarizing the internal (red) and terminal (blue) branch lengths in phylogenies derived from a real HIV-1 data set (top) and from data simulated under our first parameterization of the birth-death model. Each distribution was normalized by the mean branch length of the respective tree.
(TIFF)

S2 Fig Performance of MMPP and nonparametric clustering methods on simulated data with an extreme disparity in transmission rates. Data were simulated under the Set 1 parameterization of the birth-death model, except that the ratio of transmission rates between subpopulations was elevated from 3- to 10-fold. The *x*- and *y*-axes correspond to the false (FPR) and true positive rates (TPR) of classifying individuals into the minority subpopulation, respectively. Each point represents the outcome when the MMPP model was applied to one of 100 replicate simulations. The mean TPR and FPR for MMPP were 93% and 7.3%, respectively. We observed that the MMPP no longer suffered from a higher FPR in any replicates, which implies that the greater rate disparity prevented the MMPP model from incorrectly assigning the higher rate class at the root of the tree. Each line represents the receiver-operator characteristic curve for each of the nonparametric clustering methods (see figure legend). The nonparametric results were very similar to those obtained under a 3-fold rate disparity in transmission rates (Fig 2).
(TIFF)

S3 Fig Distribution of ML estimates of the ratio of branching rates for the two-class MMPP model. Each curve represents a kernel density summarizing the distribution of ratio estimates obtained from 100 replicate simulations for trees with *n* tips each, where *n* was varied from 100 to 1000 (see legend). Each simulation was generated from a transmission tree simulated under a birth-death in MASTER, on which sequences were simulated under a codon substitution model with INDELible and then used to reconstruct a phylogeny with rapidNJ. The true branching rate ratio is 3.0, as indicated by the dashed line.
(TIFF)

S4 Fig Performance of MMPP and five nonparametric clustering methods on simulated data with an alternate parameterization of the model (Set 2, see S1 Table). Sequence data were simulated under three scenarios where the minority subpopulation had (1) a faster transmission rate (left); (2) a faster sampling rate (centre), or; (3) both faster rates of transmission and sampling (right). The *x*- and *y*-axes correspond to the false and true positive rates of classifying individuals into the minority subpopulation, respectively. Each point represents the outcome when the MMPP model was applied to one of 100 replicate simulations. Each line represents the receiver-operator characteristic curve for one of the five nonparametric clustering methods (see figure legend), where different false and true positive rates were obtained by varying a threshold parameter of the method. Note that transmission rates in the minority subpopulation were only elevated by two-fold in this parameterization, in contrast to a three-fold increase in the first parameterization—this may partly explain an increase in false positive rates.
(TIFF)

S5 Fig Computing times required for our MMPP program to process trees with varying numbers of tips. Log-transformed computing times (*y*-axis) required for our MMPP program to process trees with varying numbers of tips. For each number of tips (*x*-axis), we simulated 10 replicate trees under the ‘faster transmission’ model. Each point represents a replicate simulation, labeled and coloured to indicate that 2 (blue) or 3 (red) rate classes were used in the model. The number of tips was varied along the following sequence: {100, 200, 500, 600, 700, 800, 900, 1000, 2000, 5000, 10000, 20000, 50000}. We used a random ‘jitter’ factor (∼ Unif(0.8, 1.2)) to separate points with respect to the *x*-axis for clarity. Our simulation experiments were concentrated in the interval between 500 and 1000 tips, where we determined that some transition in time complexity occurs. A dashed line represents a linear model fit to the computing times for *n* > 800 with two rate classes. All runs were executed on an Intel Xeon E5-1650v4 processor.
(TIFF)

S6 Fig Lineages-through-time (LTT) plot for the maximum likelihood phylogeny reconstructed from a real HIV-1 *pol* sequence data set [38]. The depth (*x*-axis) corresponds to the distance of internal nodes from the root in branch length units (expected number of nucleotide substitutions). The *y*-axis corresponding to the number of lineages is log-transformed to emphasize the location of an exponential period of growth.
(TIFF)

S7 Fig Cluster predictions from the three other nonparametric clustering methods that were not depicted in Fig 4. To obtain a similar number of clusters and individuals as reported in the original study [38], we used a TN93 cutoff of 0.6%, a patristic distance cutoff of 1%, and a phyloPart cutoff of 0.02%.
(TIFF)

S8 Fig Evaluating the effect of autocorrelated continuous rate variation. To quantify the impact of autocorrelation in branching rates on model-based clustering, we generated simulations where internal branch lengths were adjusted on a continuous scale by an evolving factor. (*left*) False positive rates under an unstructured (negative control) birth-death model. The 2-rate class MMPP model applied to simulated trees where branching rates were varied using the *simulate.autocor.kishino* function in R package NELSI [54]. The extent of rate autocorrelation and variation on a continuous scale was controlled by a single parameter that is akin to the rate of a random walk along each branch [55]. We observed a significant association between FPR and *ν* (binomial GLM, *z* = 141.4, *P* < 10^−16^; depicted by red line). (*right*) Scatterplot of internal branch lengths between trees simulated from an unstructured birth-death model (*x*-axis) and trees processed with NELSI (*y*-axis). Red points correspond to *ν* = 0.1 and black points to *ν* = 0.5.
(TIFF)

S1 Table Parameter values used for birth-death SIR model simulations. When present, the subscript indicates that the parameter is associated with the majority (*N*_0_ = 9000) or minority (*N*_1_ = 1000) subpopulation. We generated trees under two different sets of parameters, each comprising three scenarios (faster transmission, faster sampling, and both faster rates in the minority subpopulation). The first parameter set was adjusted until the model produced trees with the characteristic star-like shape of HIV-1 among-host phylogenies [16]. The second parameter set was derived from [27]; however, we increased the sampling rates relative to that study, so that the target sample size (*n* = 1000) could be obtained before every individual in the population had become infected.
(PDF)
