# Supplementary figures and images for: A model-based clustering method to detect infectious disease transmission outbreaks from sequence variation
Source: PLoS Comput Biol. 2017 Nov 13;13(11):e1005868. doi: 10.1371/journal.pcbi.1005868 (PMC5703573; doi:10.1371/journal.pcbi.1005868)

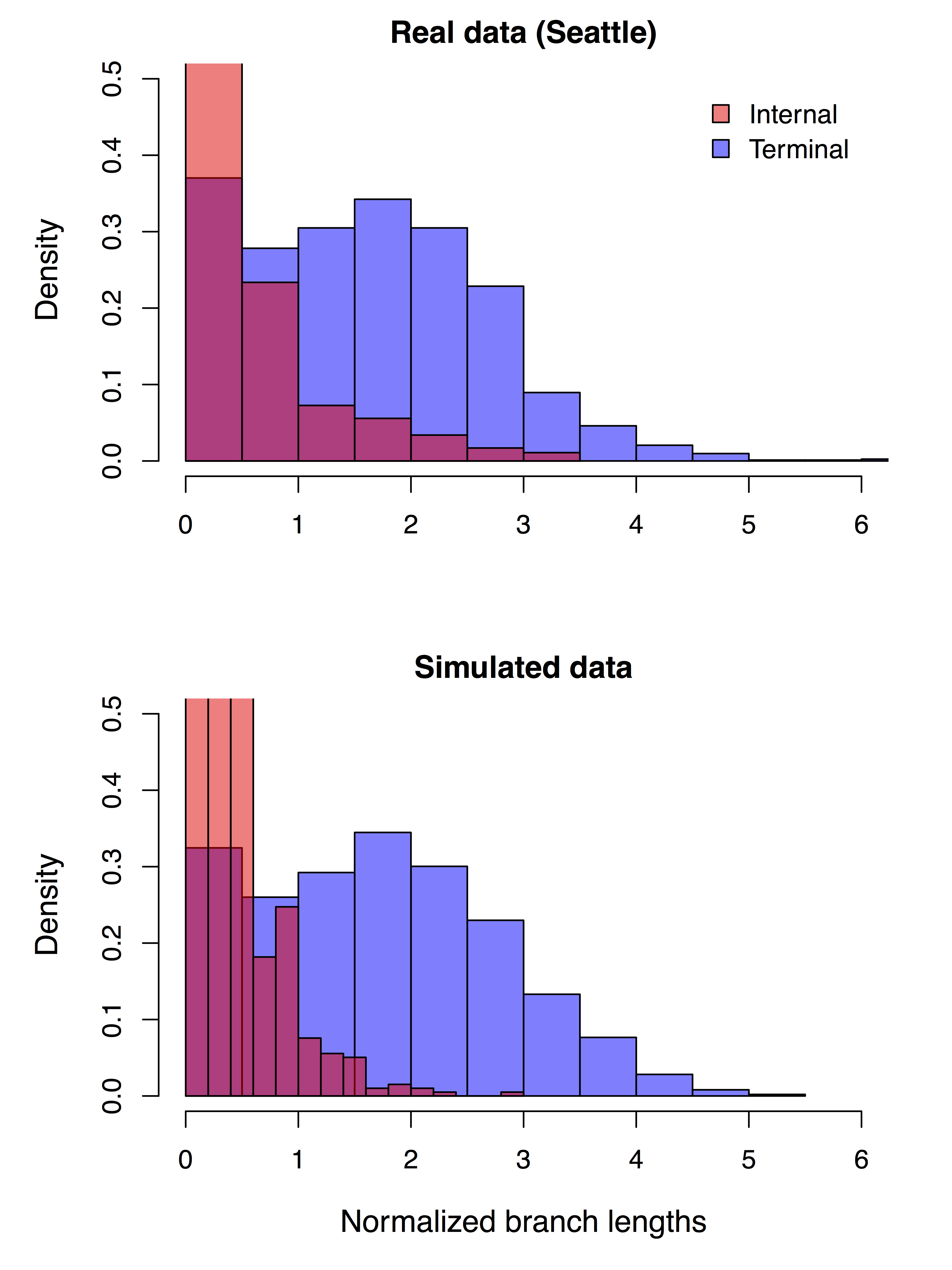

Supplement: S1 Fig — Histograms summarizing the internal (red) and terminal (blue) branch lengths in phylogenies derived from a real HIV-1 data set (top) and from data simulated under our first parameterization of the birth-death model. Each distribution was normalized by the mean branch length of the respective tree. (TIFF) [file pcbi.1005868.s001.tiff]

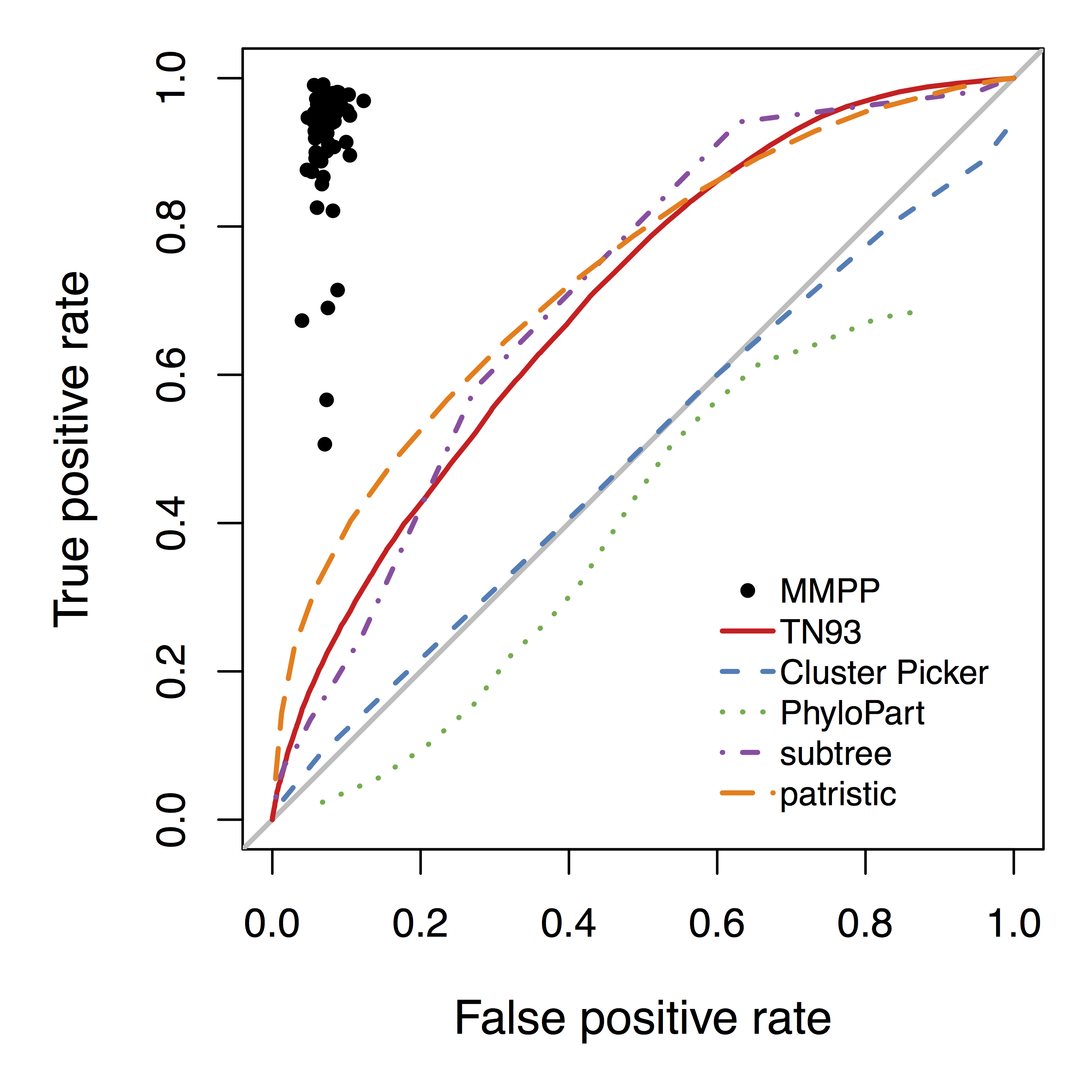

Supplement: S2 Fig — Data were simulated under the Set 1 parameterization of the birth-death model, except that the ratio of transmission rates between subpopulations was elevated from 3- to 10-fold. The x- and y-axes correspond to the false (FPR) and true positive rates (TPR) of classifying individuals into the minority subpopulation, respectively. Each point represents the outcome when the MMPP model was applied to one of 100 replicate simulations. The mean TPR and FPR for MMPP were 93% and 7.3%, respectively. We observed that the MMPP no longer suffered from a higher FPR in any replicates, which implies that the greater rate disparity prevented the MMPP model from incorrectly assigning the higher rate class at the root of the tree. Each line represents the receiver-operator characteristic curve for each of the nonparametric clustering methods (see figure legend). The nonparametric results were very similar to those obtained under a 3-fold rate disparity in transmission rates (Fig 2). (TIFF) [file pcbi.1005868.s002.tiff]

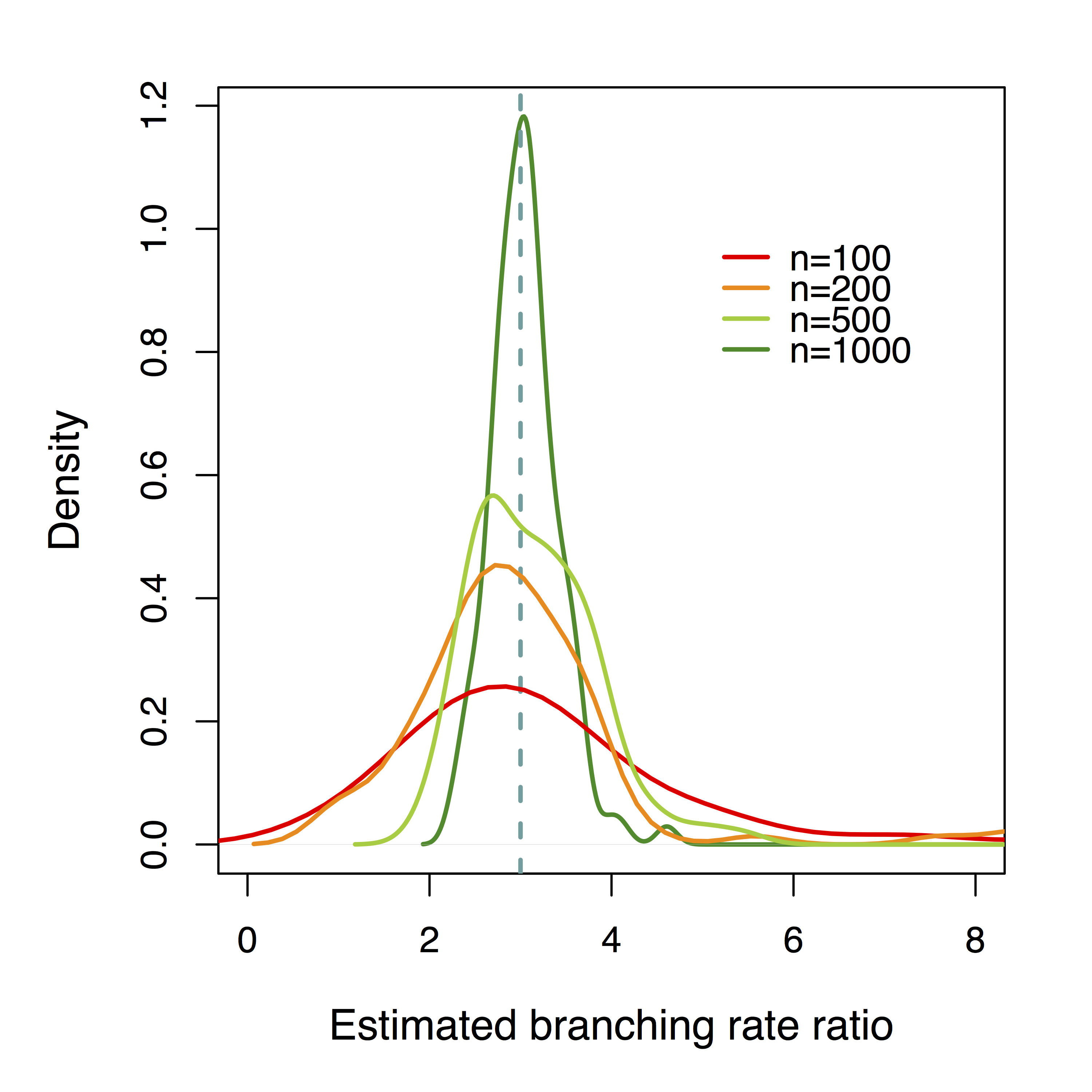

Supplement: S3 Fig — Each curve represents a kernel density summarizing the distribution of ratio estimates obtained from 100 replicate simulations for trees with n tips each, where n was varied from 100 to 1000 (see legend). Each simulation was generated from a transmission tree simulated under a birth-death in MASTER, on which sequences were simulated under a codon substitution model with INDELible and then used to reconstruct a phylogeny with rapidNJ. The true branching rate ratio is 3.0, as indicated by the dashed line. (TIFF) [file pcbi.1005868.s003.tiff]

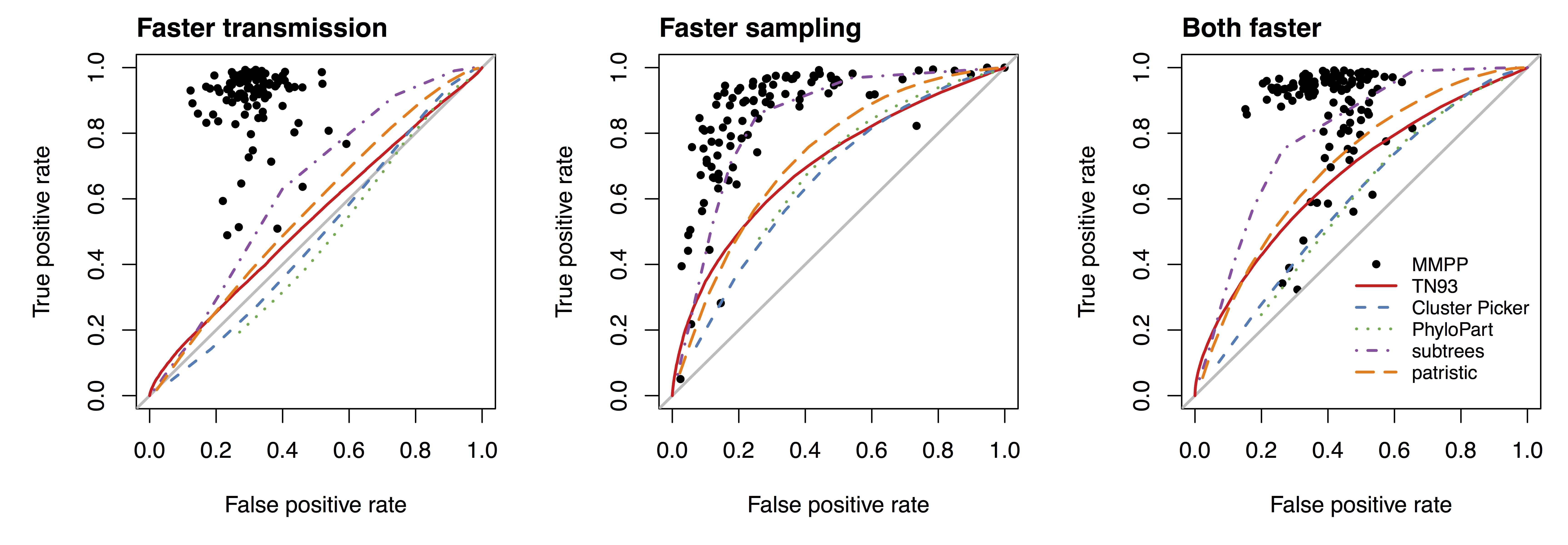

Supplement: S4 Fig — Sequence data were simulated under three scenarios where the minority subpopulation had (1) a faster transmission rate (left); (2) a faster sampling rate (centre), or; (3) both faster rates of transmission and sampling (right). The x- and y-axes correspond to the false and true positive rates of classifying individuals into the minority subpopulation, respectively. Each point represents the outcome when the MMPP model was applied to one of 100 replicate simulations. Each line represents the receiver-operator characteristic curve for one of the five nonparametric clustering methods (see figure legend), where different false and true positive rates were obtained by varying a threshold parameter of the method. Note that transmission rates in the minority subpopulation were only elevated by two-fold in this parameterization, in contrast to a three-fold increase in the first parameterization—this may partly explain an increase in false positive rates. (TIFF) [file pcbi.1005868.s004.tiff]

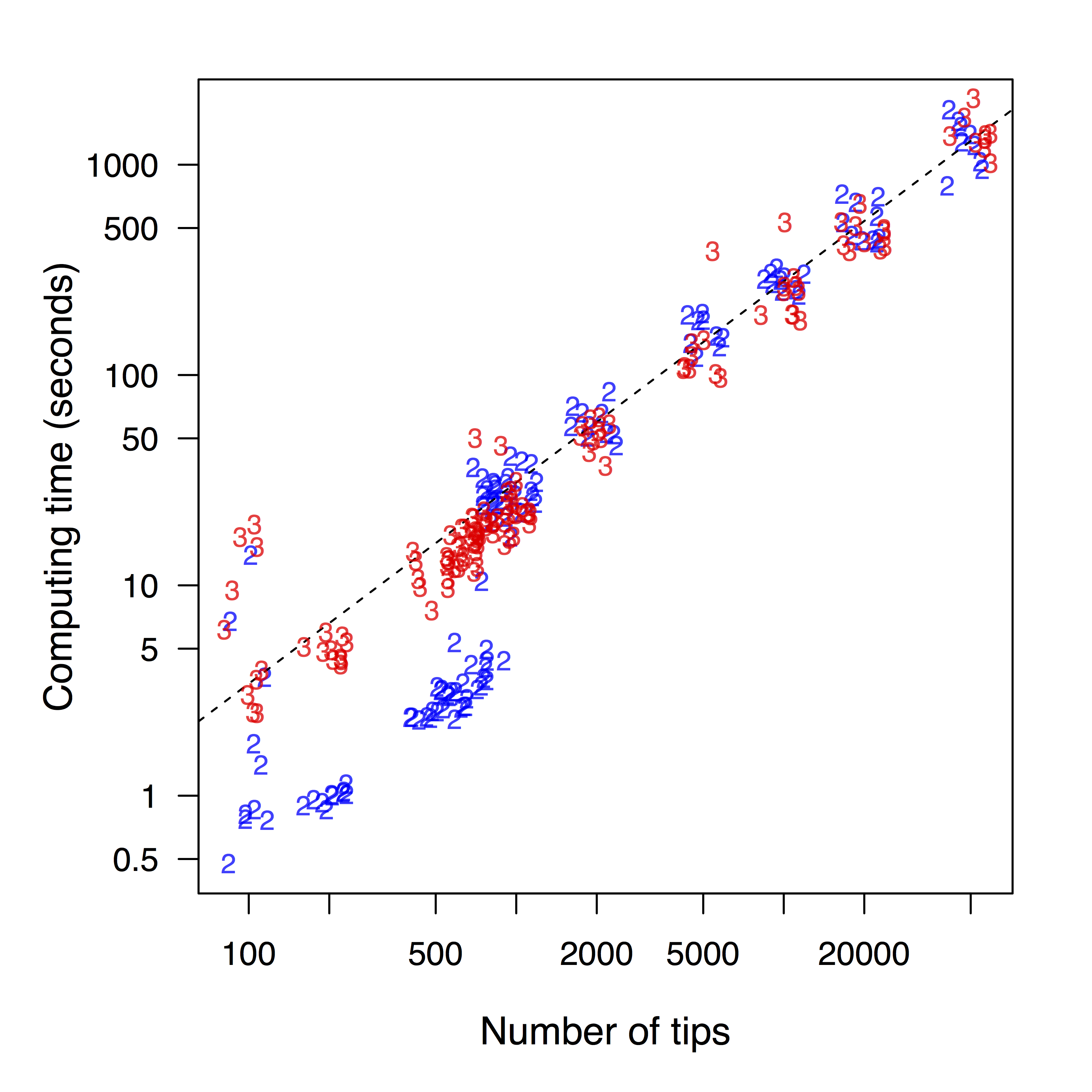

Supplement: S5 Fig — Log-transformed computing times (y-axis) required for our MMPP program to process trees with varying numbers of tips. For each number of tips (x-axis), we simulated 10 replicate trees under the ‘faster transmission’ model. Each point represents a replicate simulation, labeled and coloured to indicate that 2 (blue) or 3 (red) rate classes were used in the model. The number of tips was varied along the following sequence: {100, 200, 500, 600, 700, 800, 900, 1000, 2000, 5000, 10000, 20000, 50000}. We used a random ‘jitter’ factor (∼ Unif(0.8, 1.2)) to separate points with respect to the x-axis for clarity. Our simulation experiments were concentrated in the interval between 500 and 1000 tips, where we determined that some transition in time complexity occurs. A dashed line represents a linear model fit to the computing times for n > 800 with two rate classes. All runs were executed on an Intel Xeon E5-1650v4 processor. (TIFF) [file pcbi.1005868.s005.tiff]

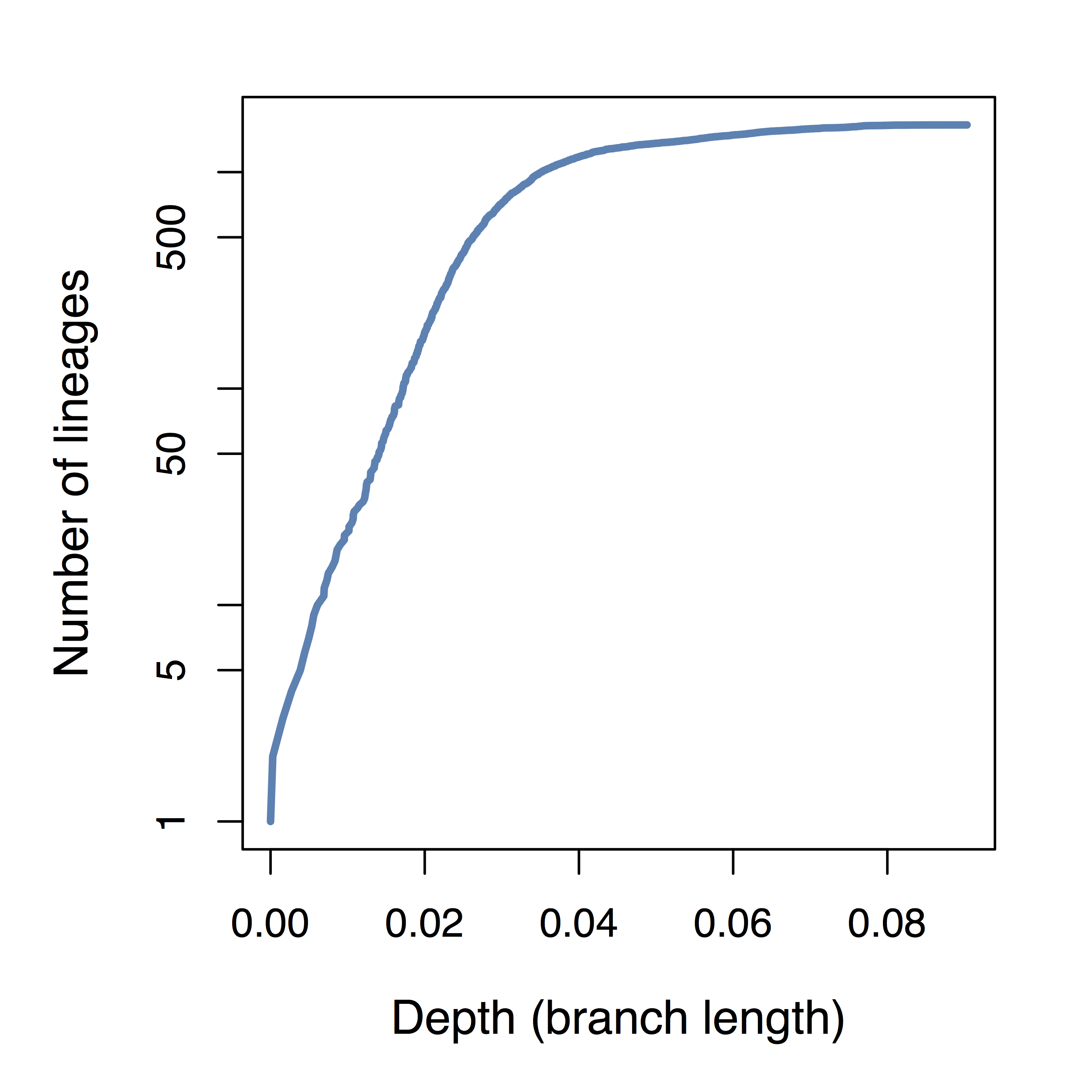

Supplement: S6 Fig — The depth (x-axis) corresponds to the distance of internal nodes from the root in branch length units (expected number of nucleotide substitutions). The y-axis corresponding to the number of lineages is log-transformed to emphasize the location of an exponential period of growth. (TIFF) [file pcbi.1005868.s006.tiff]

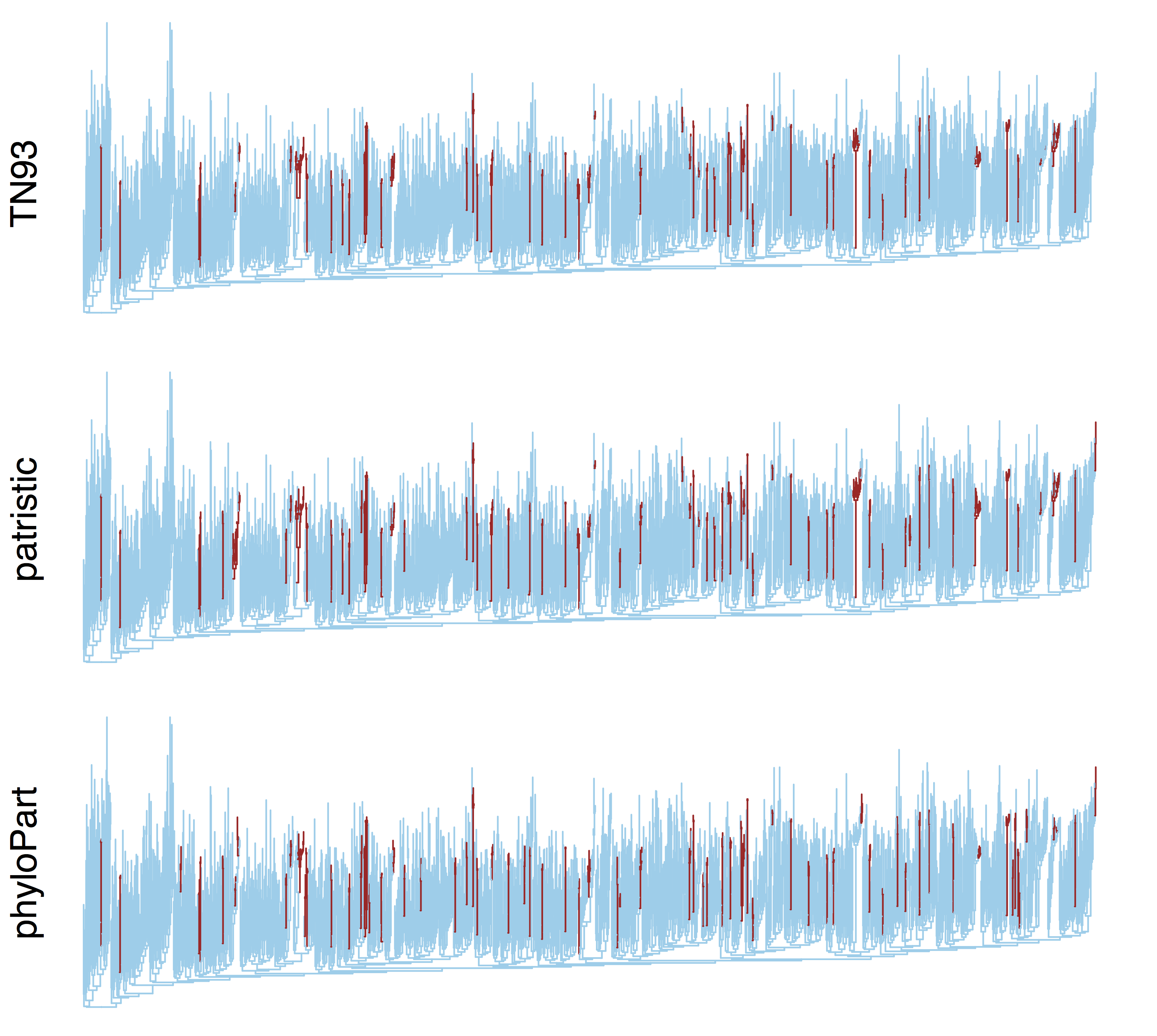

Supplement: S7 Fig — To obtain a similar number of clusters and individuals as reported in the original study [38], we used a TN93 cutoff of 0.6%, a patristic distance cutoff of 1%, and a phyloPart cutoff of 0.02%. (TIFF) [file pcbi.1005868.s007.tiff]

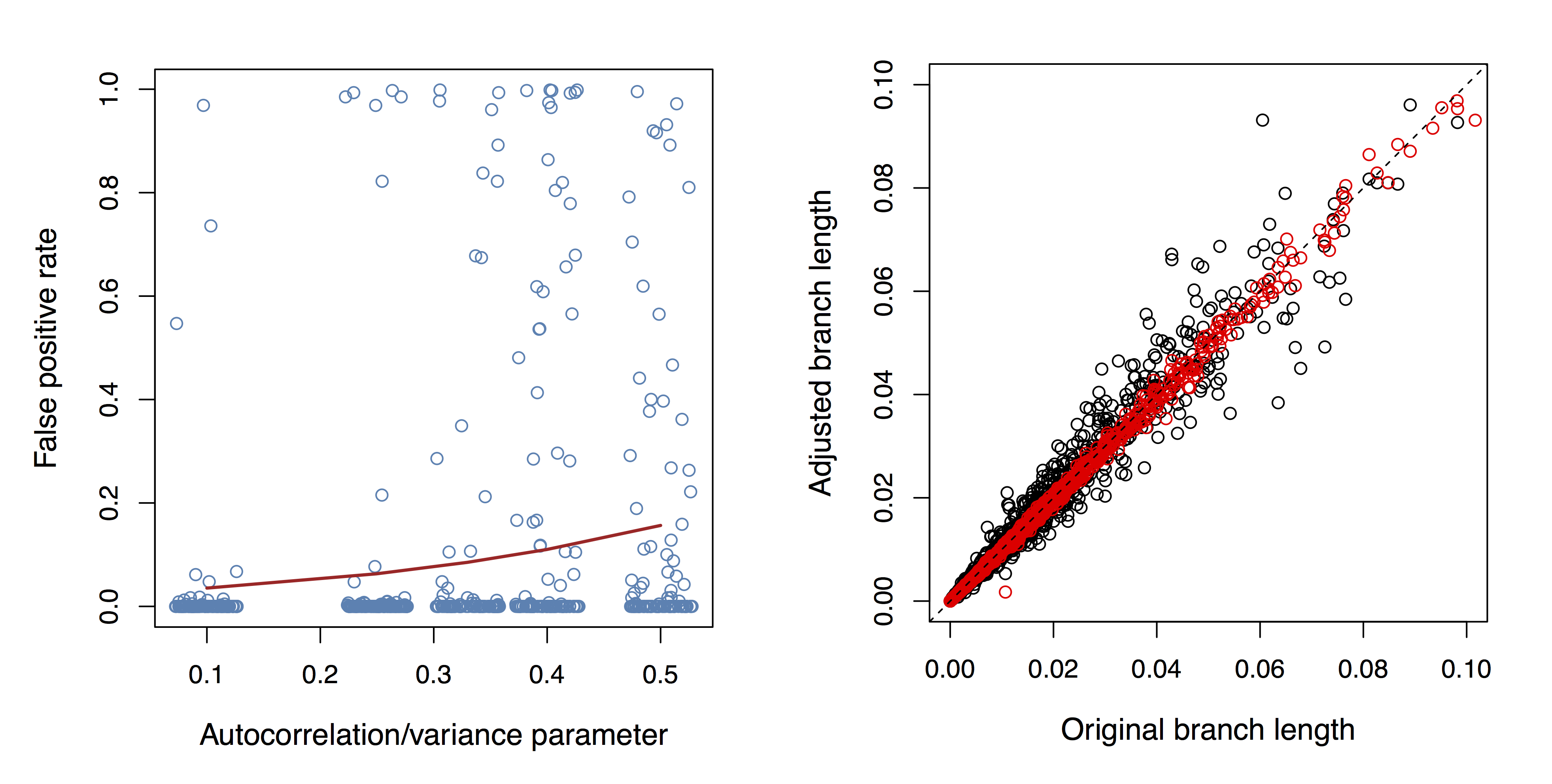

Supplement: S8 Fig — To quantify the impact of autocorrelation in branching rates on model-based clustering, we generated simulations where internal branch lengths were adjusted on a continuous scale by an evolving factor. (left) False positive rates under an unstructured (negative control) birth-death model. The 2-rate class MMPP model applied to simulated trees where branching rates were varied using the simulate.autocor.kishino function in R package NELSI [54]. The extent of rate autocorrelation and variation on a continuous scale was controlled by a single parameter that is akin to the rate of a random walk along each branch [55]. We observed a significant association between FPR and ν (binomial GLM, z = 141.4, P < 10−16; depicted by red line). (right) Scatterplot of internal branch lengths between trees simulated from an unstructured birth-death model (x-axis) and trees processed with NELSI (y-axis). Red points correspond to ν = 0.1 and black points to ν = 0.5. (TIFF) [file pcbi.1005868.s008.tiff]
