# Supplementary material for: A model-based clustering method to detect infectious disease transmission outbreaks from sequence variation
Source: PLoS Comput Biol. 2017 Nov 13;13(11):e1005868. doi: 10.1371/journal.pcbi.1005868 (PMC5703573; doi:10.1371/journal.pcbi.1005868)
Supplement: S1 Table — When present, the subscript indicates that the parameter is associated with the majority (N0 = 9000) or minority (N1 = 1000) subpopulation. We generated trees under two different sets of parameters, each comprising three scenarios (faster transmission, faster sampling, and both faster rates in the minority subpopulation). The first parameter set was adjusted until the model produced trees with the characteristic star-like shape of HIV-1 among-host phylogenies [16]. The second parameter set was derived from [27]; however, we increased the sampling rates relative to that study, so that the target sample size (n = 1000) could be obtained before every individual in the population had become infected. (PDF) [file pcbi.1005868.s009.pdf]

| **Rate parameter** | | **Set 1** | | | **Set 2** | | |
|---|---|---|---|---|---|---|---|
| | | Fast trans. | Fast samp. | Both | Fast trans. | Fast samp. | Both |
| Transmission | $\beta_0$ | 0.005 | 0.005 | 0.005 | $7.5 \times 10^{-4}$ | $7.5 \times 10^{-4}$ | $7.5 \times 10^{-4}$ |
| | $\beta_1$ | 0.135 | 0.045 | 0.135 | 0.0135 | $6.75 \times 10^{-3}$ | 0.0135 |
| Migration | $m$ | 0.05 | 0.05 | 0.05 | 0.05 | 0.05 | 0.05 |
| Mortality | $\mu$ | 0.01 | 0.01 | 0.01 | 1.0 | 1.0 | 1.0 |
| Sampling | $\psi_0$ | 0.5 | 0.5 | 0.5 | 1.0 | 1.0 | 1.0 |
| | $\psi_1$ | 0.5 | 2.5 | 2.5 | 1.0 | 2.0 | 2.0 |

Table S1: Parameter values used for birth-death SIR model simulations.
